# Predicting Morbidity in Pancreaticoduodenectomy: A Focus on Nutritional Status

**DOI:** 10.7759/cureus.109211

**Published:** 2026-05-19

**Authors:** Sadaf Ali, Mohammad Younis Bhat, Mohd Riyaz Lattoo, Akashdeep S Sohi, Ajay Vane

**Affiliations:** 1 Surgical Gastroenterology, Sher-i-Kashmir Institute of Medical Sciences, Srinagar, IND; 2 Surgical Gastroenterology, Dr. D. Y. Patil Medical College, Hospital and Research Centre, Dr. D. Y. Patil Vidyapeeth, Pune (Deemed to be University), Pune, IND

**Keywords:** nutritional status, pancreatic fistula, pancreaticoduodenectomy, postoperative morbidity, surgical site infection

## Abstract

Background and aim

Pancreaticoduodenectomy (Whipple procedure) remains associated with high postoperative morbidity, including surgical site infections (SSIs) and pancreatic fistulas, despite advances in surgical care. Although the role of nutrition in pancreatic surgery is increasingly recognized, prospective data evaluating multiple nutritional indices in predicting postoperative morbidity remain limited. This study investigates the association between preoperative nutritional status and postoperative complications following pancreaticoduodenectomy.

Methods

A prospective observational study enrolled 164 patients undergoing pancreaticoduodenectomy at a tertiary care institute in the Jammu and Kashmir region of India from January 2020 to December 2024. Nutritional status was assessed preoperatively using serum albumin levels, the Malnutrition Universal Screening Tool (MUST), the Nutritional Risk Index (NRI), and the percentage of weight loss. Primary outcomes were SSI incidence (per the CDC criteria) and pancreatic fistula grade (per the International Study Group on Pancreatic Surgery 2016). Univariate and multivariate logistic regression analyses identified predictors, with propensity score matching to address bias; p < 0.05 was considered significant.

Results

Patients had a mean age of 55 years (range: 32-78), with 55% male and 97.6% of cases being malignant (84.8% adenocarcinoma). Univariate analysis showed significant associations (all p < 0.001) between poor nutritional parameters, such as severe hypoalbuminemia (<2.5 g/dL), high MUST/NRI scores, and >5% weight loss, and increased SSI/pancreatic fistula rates. Multivariate analysis confirmed these as independent risk factors.

Conclusions

Preoperative malnutrition strongly predicts postoperative SSIs and pancreatic fistulas after pancreaticoduodenectomy. Routine nutritional screening with MUST, NRI, albumin, and weight loss assessment, followed by prehabilitation, can optimize outcomes in this high-risk population.

## Introduction

Pancreaticoduodenectomy (Whipple procedure) is a complex surgical intervention commonly performed for the management of periampullary, pancreatic head, and uncinate neoplasms. Despite advancements in surgical techniques and perioperative care, it remains associated with considerable postoperative morbidity. Among the most frequently encountered complications are surgical site infections (SSIs), postoperative pancreatic fistulas (POPFs), delayed gastric emptying, and dumping syndrome, all of which adversely affect patient recovery and long-term outcomes [1].

SSI is a leading cause of postoperative morbidity and can significantly prolong hospitalization, increase healthcare expenditures, and reduce overall quality of life [2]. Similarly, pancreatic fistulas are a common and clinically significant complication, often resulting in malnutrition, fluid and electrolyte imbalances, and delayed return to baseline physiological function [3].

Identification of reliable predictors of postoperative morbidity is critical to enhancing clinical outcomes and guiding perioperative management. Several studies have highlighted the impact of factors such as preoperative nutritional status, existing comorbidities, and operative duration on postoperative outcomes following pancreaticoduodenectomy [4,5]. However, the interplay between these variables and their cumulative effect on morbidity remains incompletely elucidated.

Preoperative nutritional status has emerged as a key determinant of surgical outcomes. Malnutrition in surgical patients is defined as a state of deficient energy, protein, or micronutrients that impairs tissue repair and immune function. It can be objectively evaluated using tools such as the Malnutrition Universal Screening Tool (MUST), the Nutritional Risk Index (NRI), serum albumin levels [6], and percentage of preoperative weight loss. The MUST score provides a standardized approach to identifying malnourished individuals [7], while the NRI incorporates parameters such as serum albumin, total lymphocyte count, and triceps skinfold thickness [8]. Additionally, the percentage of weight loss serves as a surrogate marker of nutritional decline, calculated as the patient's weight loss divided by their preoperative weight [9].

The present study aims to evaluate whether preoperative nutritional parameters independently predict postoperative morbidity following pancreaticoduodenectomy and to compare the clinical utility of serum albumin, MUST score, NRI score, and percentage weight loss in identifying patients at increased risk for SSI and POPF.

## Materials and methods

This prospective observational study was conducted over a five-year period, from January 2020 to December 2024, at a tertiary care academic institute in Jammu and Kashmir, India. The study included 164 patients who underwent pancreaticoduodenectomy (Whipple procedure) during this time frame. Inclusion criteria included all adult patients undergoing pancreaticoduodenectomy for malignant or benign periampullary lesions during the study period. Exclusion criteria included patients undergoing palliative bypass procedures, emergency pancreatic resections, patients with incomplete nutritional or follow-up data, and patients lost to follow-up within 30 postoperative days. All eligible consecutive patients undergoing pancreaticoduodenectomy during the study period who fulfilled the inclusion criteria were prospectively enrolled. The sample size was determined based on consecutive patient enrollment during the study period. Due to the observational design and limited institutional pancreatic surgery volume, convenience sampling was employed. The final sample size of 164 patients was considered adequate to evaluate the association between preoperative nutritional parameters and postoperative morbidity.

Comprehensive clinical and pathological data were collected for each patient, encompassing demographic variables (age and gender), smoking history, preoperative diagnosis (indication for surgery), presence or absence of preoperative biliary stenting, tumor location, and nutritional parameters. Nutritional status was assessed preoperatively via serum albumin levels, the MUST, the NRI, and percentage weight loss, calculated based on unintentional body weight reduction during the six months preceding surgery.

The primary postoperative outcomes evaluated were the incidence of SSIs and the grade of POPF. SSIs were defined in accordance with the CDC criteria as infections occurring within 30 days postoperatively, including superficial and deep wound infections and intra-abdominal abscesses. Pancreatic fistula was defined and classified according to the International Study Group on Pancreatic Surgery (ISGPS) 2016 grading system [10].

Statistical analysis was performed using IBM SPSS Statistics for Windows, version 22.0 (released 2013; IBM Corp., Armonk, NY, USA). All laboratory values were analyzed as continuous variables. Student’s t-test and ANOVA were performed for variables with a normal distribution. Mann-Whitney U tests and Kruskal-Wallis tests were conducted for variables with non-normal distributions. Categorical data, described as frequencies with percentages, were compared using Pearson’s chi-square test. Propensity score matching was performed to reduce selection bias and adjust for confounding variables. Variables entered into the propensity score model included age, sex, smoking history, biliary stenting status, tumor location, diagnosis, and baseline nutritional parameters. A p-value of < 0.05 was considered statistically significant. Univariate and multivariate logistic regression analyses were used to determine risk factors for postoperative complications; variables with p-values < 0.05 in univariate analysis were included in the multivariate logistic regression model.

## Results

Table 1 summarizes the clinicopathological characteristics of the study population. The age of patients ranged from 32 to 78 years, with a mean age of 55 years. Of the 164 patients, 21 (12.8%) were under 40 years of age, 131 (79.9%) were between 40 and 65 years of age, and 12 (7.3%) were older than 70 years. The study cohort consisted of 90 males (54.9%) and 74 females (45.1%). A history of smoking was present in 78 patients (47.6%), while 86 patients (52.4%) were nonsmokers.

**Table 1:** Clinicopathological characteristics of the study population NET, neuroendocrine tumor

Parameter	Category	No. of patients (n)	Percentage (%)
Age in years	<40	21	12.8%
	40-70	131	79.9%
	>70	12	7.3%
Gender	Male	90	54.9%
	Female	74	45.1%
Habit	Smoker	78	47.6%
	Nonsmoker	86	52.4%
Reason for pancreaticoduodenectomy	Malignancy	160	97.6%
	Nonmalignant	4	2.4%
Stenting	Done	103	62.8%
	Not done	61	37.2%
Nature of disease	Adenocarcinoma	139	84.8%
	NET	21	12.8%
	Nonmalignant	4	2.4%
Location of tumor	Distal cholangiocarcinoma	86	52.4%
	Ampulla	25	15.2%
	Pancreatic head	41	25%
	Duodenal tumor	8	4.9%

Malignancy was the primary indication for pancreaticoduodenectomy in 160 patients (97.6%), with adenocarcinoma being the most prevalent histological subtype, identified in 139 cases (84.8%). Four patients (2.4%) underwent the procedure for a nonmalignant indication.

Univariate analysis, as presented in Table 2, demonstrated that several parameters of preoperative nutritional status were significantly associated with postoperative complications, specifically SSIs and pancreatic fistula formation.

**Table 2:** Nutrition parameter and complications association Fistula grade was classified according to the ISGPS 2016 POPF grading system [10]. ISGPS, International Study Group on Pancreatic Surgery; MUST, Malnutrition Universal Screening Tool; NRI, Nutritional Risk Index; POPF, postoperative pancreatic fistula; SSI, surgical site infection

Outcome	Parameters	SSI	NO SSI	p-Value	Fistula Grade A	Fistula Grade B+C	No fistula	p-Value
Preoperative albumin status (g/dL)	Severe hypoalbuminemia (<2.5)	36	5	χ²(2, n = 134) = 64.357, p = 1.06 × 10⁻¹⁴	35	6	0	χ²(4, n = 164) = 91.272, p = 7.07 × 10⁻¹⁹
	Mild hypoalbuminemia (2.5-3.5)	26	36		8	16	38	
	Normal (>3.5)	5	56		4	13	44	
MUST score [7]	Low risk (0)	14	43	χ²(2, n = 164) = 23.294, p = 8.74 × 10⁻⁶	5	8	44	χ²(4, n = 164) = 77.004, p = 7.51 × 10⁻¹⁶
	Medium risk (-1)	13	32		7	4	34	
	High risk (2 or more)	40	22		35	23	4	
NRI score [8]	No malnutrition (>100)	5	45	χ²(3, n = 164) = 56.058, p = 4.08 × 10⁻¹²	4	4	42	χ²(6, n = 164) = 46.583, p = 2.27 × 10⁻⁸
	Mild (97.5-100)	8	24		8	6	18	
	Moderate (83.5-97.5)	10	16		12	4	10	
	Severe (<83.5)	44	12		23	21	12	
Percentage of weight loss	<5%	8	54	χ²(2, n = 164) = 39.682, p = 2.42 × 10⁻⁹	4	6	52	χ²(4, n = 164) = 77.243, p = 6.68 × 10⁻¹⁶
	5-10%	21	27		11	9	28	
	>10%	38	16		32	20	2	

Preoperative serum albumin levels were significantly predictive of both SSI (p < 0.001) and fistula (p < 0.001). The MUST score showed a strong association with SSI (p < 0.001) and fistula (p < 0.001). The NRI was also a significant predictor of SSI (p < 0.001) and fistula (p < 0.001). Additionally, the percentage of preoperative weight loss demonstrated a statistically significant association with SSI (p < 0.001) and fistula (p < 0.001).

These findings reinforce the prognostic significance of preoperative nutritional status in predicting postoperative morbidity and align with existing literature emphasizing its critical role in improving surgical outcomes and reducing complications following major gastrointestinal procedures.

## Discussion

The high prevalence of malnutrition among surgical patients further compounds the complexity of pancreaticoduodenectomy (Whipple procedure) [11]. This study aims to highlight the significant correlation between preoperative nutritional status and postoperative morbidity, stressing the necessity for early detection and enhancement of modifiable risk factors to improve surgical outcomes. Nutritional status, as assessed through the MUST, the NRI, serum albumin levels, and percentage of unintentional weight loss, emerged as a significant predictor of postoperative complications.

Serum albumin has long been established as a surrogate marker for nutritional status and a prognostic indicator of surgical risk. Serum albumin is one of the most widely studied biochemical markers in surgical risk prediction [12]. Albumin reflects not only nutritional reserves but also systemic inflammatory and hepatic synthetic function. Low albumin levels impair collagen synthesis, reduce fibroblast proliferation, and weaken neovascularization at the surgical site. Furthermore, low serum albumin reduces plasma colloid osmotic pressure, causing interstitial fluid accumulation and tissue edema. This edema impairs microvascular perfusion and delays wound healing. It also weakens local immune cell function, increasing susceptibility to infection. The protein-rich fluid in edematous tissues further supports bacterial growth. Collectively, these factors make hypoalbuminemic patients more prone to developing SSI after major abdominal surgery [13-15]. From a pancreatic anastomosis perspective, albumin contributes to tissue tensile strength and integrity of the pancreaticoenteric anastomosis. Reduced albumin is known to correlate with increased tissue edema and fragility, which may predispose to POPF, particularly in patients with a soft pancreatic texture [16]. In our cohort, hypoalbuminemia was significantly associated with both SSIs (p < 0.001) and pancreatic fistula formation (p < 0.001). These findings are consistent with prior studies by Tfaily et al. and Chai and Jiffre [17,18], which emphasized the value of preoperative serum albumin assessment in surgical planning and risk reduction.

Malnutrition in general can be assessed by different tools and risk scores such as MUST, NRI, and percentage weight loss. It has a multifactorial impact on postoperative infection risk. Malnourished patients exhibit reduced cell-mediated immunity, including impaired T-lymphocyte function, decreased complement activity, and reduced phagocytic function, as well as decreased subcutaneous tissue oxygen tension, compromising neutrophil action and delaying epithelial regeneration. These immune and metabolic deficits create a permissive environment for SSI development [19-21]. Unintentional preoperative weight loss is a surrogate for chronic hypermetabolism, tumor-induced catabolism, and inadequate caloric/protein intake. Weight loss reflects a cumulative decline in lean body mass, including visceral protein stores required for collagen synthesis, and correlates with decreased immune function [22,23]. Patients with significant preoperative weight loss and high nutritional risk scores likely have diminished protein reserves, decreased micronutrient stores (zinc and vitamin C), and impaired fibroblast activity. These parameters are critical for strengthening the anastomosis, wound healing, and promoting enzymatic containment during the early postoperative phase [24,25].

In our study, we evaluated the MUST score and the NRI score. The MUST score, a validated screening tool incorporating body mass index, recent weight loss, and acute disease impact, demonstrated strong predictive value. Patients with higher MUST scores exhibited significantly greater rates of SSI (p < 0.001) and POPF (p < 0.001). These results are supported by recent literature, including the work of La Torre et al. and Stratton et al. [26,27], reinforcing the clinical utility of MUST in identifying malnourished patients at higher risk for adverse surgical outcomes.

Similarly, the NRI score, which integrates serum albumin, total lymphocyte count, and anthropometric measures, was found to be a significant predictor of postoperative morbidity. Elevated NRI scores were associated with increased risk of SSI (p < 0.001) and fistula (p < 0.001), in alignment with findings reported by Shinkawa et al. [28], further substantiating the role of comprehensive nutritional risk stratification in perioperative care.

In addition to these indices, the percentage of unintentional preoperative weight loss was independently associated with higher complication rates. Patients who experienced greater weight loss preoperatively were significantly more likely to develop SSI (p < 0.001) and fistula (p < 0.001). These results parallel observations by Thirunavukarasu et al. [29], who have demonstrated that weight loss adversely impacts wound healing and immune competence, leading to heightened postoperative morbidity.

Collectively, these findings reinforce the clinical importance of preoperative nutritional status in pancreatic surgery and suggest that malnutrition may represent a potentially modifiable risk factor with direct implications for surgical outcomes and demonstrate the value of prehabilitation in pancreatic surgery; however, interventional studies are required to determine whether nutritional optimization directly improves postoperative outcomes. The integration of nutritional screening and targeted preoperative interventions, such as dietary counseling, enteral supplementation, or immunonutrition, may reduce complication rates and enhance recovery following Whipple procedures.

Our findings support implementing the following strategies in the clinical pathway for patients undergoing pancreaticoduodenectomy: (1) routine preoperative nutritional screening using albumin, MUST, NRI, and weight-loss percentage; (2) categorization of “nutritional high-risk” patients, prompting a prehabilitation protocol of seven to 14 days; (3) integration of dietitians into the multidisciplinary pancreatic cancer team; and (4) postoperative nutritional optimization, including enteral feeding as early as possible.

An additional factor influencing nutritional and postoperative outcomes in patients undergoing pancreaticoduodenectomy is obstructive jaundice, particularly in pancreatic head and periampullary malignancies. Jaundice may contribute to impaired nutritional status through anorexia, malabsorption, altered hepatic synthetic function, and immune dysfunction, thereby potentially increasing postoperative morbidity. Patients presenting with obstructive jaundice may therefore require even more meticulous preoperative optimization, as jaundice can further impair nutritional status, wound healing, and immune response following major pancreatic surgery.

Limitations

Our study has several limitations that should be acknowledged. First, this was a single-center prospective observational study, which may limit the generalizability of the findings to other populations and healthcare settings. Second, no external validation cohort was included to validate the predictive utility of the nutritional parameters assessed. Third, postoperative outcome assessors were not blinded, which may have introduced detection bias. In addition, although multivariate analysis and propensity score matching were performed to reduce confounding, residual confounding inherent to observational studies cannot be completely excluded. Furthermore, nutritional status was assessed at a single preoperative time point, and the study did not evaluate whether targeted nutritional interventions could modify postoperative outcomes. Future multicenter studies with larger cohorts and interventional designs are warranted to further validate these findings.

## Conclusions

This study demonstrates the significant impact of preoperative nutritional status on the risk of postoperative complications, particularly SSIs and pancreatic fistulas, in patients undergoing pancreaticoduodenectomy. The findings confirm that malnutrition, assessed through serum albumin levels, MUST score, NRI score, and percentage of weight loss, is closely associated with increased postoperative morbidity. SSIs contribute substantially to postoperative morbidity, prolonged hospital stay, and delayed recovery, while POPFs are associated with significant morbidity and may also contribute to increased postoperative mortality. These results support the integration of thorough nutritional assessment into the preoperative evaluation and emphasize the clinical value of nutritional optimization in improving surgical outcomes in this high-risk patient population. However, further prospective, multicenter, and interventional studies are required to determine whether active preoperative nutritional optimization can directly reduce postoperative morbidity and improve clinical outcomes.
